# Income Insufficiency, Type 2 Diabetes, and Perceived Stress in Cardiac Arrhythmia Outpatients: A Cross-Sectional Study

**DOI:** 10.3390/healthcare14142197

**Published:** 2026-07-20

**Authors:** Fahad Alsaikhan

**Affiliations:** Department of Clinical Pharmacy, College of Pharmacy, Prince Sattam Bin Abdulaziz University, P.O. Box 173, Alkharj 11942, Saudi Arabia; fsaikhan@hotmail.com; Tel.: +966-11-588-6074

**Keywords:** atrial fibrillation, cardiac arrhythmia, stress, psychological, psychometrics, type 2 diabetes mellitus, Saudi Arabia

## Abstract

**Background and Objectives:** Atrial fibrillation (AF) and related arrhythmias carry a substantial psychosocial burden that current cardiology guidelines do not systematically address. Whether economic strain and type 2 diabetes mellitus (T2DM) cluster with high perceived stress in arrhythmia outpatients remains untested in Arab populations. The objective of this study was to estimate the prevalence of high perceived stress and to identify its independent sociodemographic and clinical correlates in Saudi outpatients with cardiac arrhythmia, with an a priori focus on income insufficiency and type 2 diabetes mellitus. **Materials and Methods:** Between December 2025 and January 2026, 282 adults with clinician-documented arrhythmia attending a tertiary cardiology clinic in Saudi Arabia completed a structured questionnaire including an Arabic 10-item Perceived Stress Scale (PSS-10) previously validated in Arabic-speaking populations. High perceived stress was defined as a PSS-10 score of 20 or higher. A theoretically specified multivariable logistic regression with Firth penalisation identified independent correlates. Three sensitivity analyses were prespecified, and the latent structure of the Arabic PSS-10 was examined by split-sample exploratory and confirmatory factor analysis. Reporting follows the Strengthening the Reporting of Observational Studies in Epidemiology (STROBE) statement. **Results:** Sixty-nine participants (24.5%; 95% confidence interval [CI], 19.7–29.9) met the high-stress threshold (mean PSS-10 15.5; standard deviation [SD] 9.1). Income insufficiency (adjusted odds ratio [aOR] 2.09; 95% CI, 1.16–3.78; *p* = 0.014) and T2DM (aOR 2.51; 95% CI, 1.39–4.55; *p* = 0.002) were independently associated with high perceived stress; age and sex were not. The Arabic PSS-10 showed a two-factor structure (62.8% common variance) with acceptable confirmatory fit (root mean square error of approximation [RMSEA] = 0.078; 90% CI, 0.058–0.098; comparative fit index [CFI] = 0.945; Tucker–Lewis index [TLI] = 0.927; standardised root mean square residual [SRMR] = 0.052) and excellent reliability (Cronbach’s α = 0.92). **Conclusions:** Roughly one in four Saudi outpatients with cardiac arrhythmia reports high perceived stress. The burden concentrates among those with income insufficiency or T2DM. Brief PSS-10 screening paired with onward referral to financial-navigation and diabetes-distress support warrants prospective evaluation as a low-cost, scalable component of routine arrhythmia care, with longitudinal designs needed to establish temporal sequence and intervention efficacy.

## 1. Introduction

Cardiac arrhythmia is rarely a single illness in any clinically meaningful sense. AF and its close relatives drive a heavy burden of stroke, heart failure, hospitalisation, and premature mortality across high-, middle-, and low-income settings [1,2,3], and contemporary guidelines lean on rhythm and rate control alongside anticoagulation, with careful management of cardiometabolic risk completing routine cardiology care [2,3,4]. What guidelines accommodate less directly is the psychosocial dimension of arrhythmia care, where the unpredictability of palpitations sits alongside a constant low-level vigilance for complications. The clinical question that follows is a practical one. Which patients in an arrhythmia clinic carry the heaviest psychosocial load, and which routinely collected characteristics identify them at the bedside? Answering that question requires more than a generic acknowledgement that stress matters in cardiology. It requires data on which patients are most affected and through which co-occurring burdens they become so.

The scale of the problem in the Eastern Mediterranean Region merits closer attention. Over recent decades, AF incidence and prevalence in the region have risen sharply, outpacing many high-income settings [5]. Saudi Arabia sits within a broader regional shift, in which lifestyle changes and rising cardiometabolic comorbidity, set against an ageing demographic profile, have together produced a steeper trajectory than in many high-income settings. Within Saudi cohorts, hypertension affects roughly 30% of adults, diabetes about 25%, and dyslipidaemia about 32%, with substantial socioeconomic gradients [6]. The clinical implication is straightforward. Saudi cardiology clinics increasingly see arrhythmia patients carrying multiple chronic conditions on a backdrop of resource constraint, and the psychosocial consequences of this multimorbidity have not been formally measured in the population they serve.

Chronic stress is linked to adverse cardiovascular outcomes through autonomic, neuroendocrine, inflammatory, and behavioural pathways [7,8,9]. For the cardiologist, however, the more pressing implication is operational: perceived stress shapes medication adherence and symptom interpretation and conditions long-term engagement with follow-up, which makes it actionable at the clinic visit irrespective of any direct arrhythmogenic effect.

The descriptive literature on psychological morbidity in AF has grown without resolving which social and clinical factors concentrate high perceived stress in outpatient cardiology. The evidence remains fragmented. Pooled prevalence estimates of 24.3% for depression and 14.5% for anxiety in AF cohorts [10], a documented mental-health burden in nationwide European registries linked to higher stroke and mortality risk [11] and elevated stress in treated AF patients [12] together demonstrate the magnitude of the problem but not its predictors. Cohort and meta-analytic studies have linked psychosocial exposures, among them life events, depressive symptoms, insomnia, and trait anxiety, to incident AF [13,14,15]. Taken together, these findings indicate that psychological morbidity is common in AF but unevenly distributed. The question is no longer whether psychosocial factors matter for arrhythmia care but how their effect varies across subgroups and clinical contexts.

Stress-process theory predicts that chronic strain concentrates where resources are constrained rather than distributing evenly across patients [16], a pattern consistent with Saudi and international evidence linking economic disadvantage to cardiovascular risk and to perceived stress [17,18,19]. Two determinants are therefore of particular interest in the arrhythmia clinic, because both are clinically actionable and routinely recorded at intake. Income insufficiency has been linked to higher mortality and stroke risk in AF cohorts [20]. Type 2 diabetes imposes a continuous self-management burden associated with worse self-care and glycaemic outcomes [21,22] and is highly prevalent in Saudi arrhythmia populations. Where economic constraint and diabetes coexist, the likely consequence is perceived overload alongside reduced coping capacity.

Measuring perceived stress in this population requires an instrument that captures appraisal-based experience rather than a checklist of stressful events. The Lazarus and Folkman transactional model defines stress as the appraisal of demands relative to perceived coping resources [23], a definition particularly well suited to arrhythmia care, where uncertainty about recurrence and the complexity of long-term medication regimens produce continuous re-appraisal of personal control. The 10-item Perceived Stress Scale (PSS-10) operationalises this construct as perceived unpredictability and overload [24], and is conceptually distinct from depression and anxiety inventories that measure clinical syndromes. Two-factor structure approximating perceived distress and perceived coping has been documented in Western and non-Western samples [25,26], although the latent dimensionality of the Arabic PSS-10 in cardiac populations remains unestablished. Examining its structure in the target population is therefore methodologically necessary before subscale-level inferences can be drawn from group comparisons.

Three gaps therefore remain in the present clinical and methodological landscape. First, the determinants of high perceived stress in outpatient arrhythmia care have not been systematically characterised in the Saudi population, despite a decade of expanding cardiometabolic epidemiology. Second, the independent contributions of income insufficiency and T2DM to stress appraisal have not been jointly evaluated in this clinical context. Third, the psychometric properties of the Arabic PSS-10 in cardiac patients have not been established. Against this background, the present study estimated perceived stress in Saudi outpatients with cardiac arrhythmia using the Arabic PSS-10 and identified its independent sociodemographic and clinical correlates, with a priori focus on income insufficiency and T2DM. A secondary aim was to evaluate the latent structure and reliability of the instrument through a split-sample exploratory and confirmatory factor analysis design. We hypothesised that, after multivariable adjustment, both income insufficiency and T2DM would be independently associated with high perceived stress, while age and sex would not retain independent significance once these underlying determinants were accounted for.

## 2. Materials and Methods

### 2.1. Study Design and Setting

A single-centre analytical cross-sectional study was conducted between December 2025 and January 2026 at the outpatient cardiology service of a tertiary hospital affiliated with Prince Sattam Bin Abdulaziz University in Alkharj, Saudi Arabia. Reporting follows the Strengthening the Reporting of Observational Studies in Epidemiology (STROBE) statement for cross-sectional studies [27]. The completed STROBE checklist is provided as Supplementary File S1.

### 2.2. Ethical Approval

The study protocol was approved by the Scientific Research Ethics Committee at Prince Sattam Bin Abdulaziz University (approval number SCBR-614/2025). All procedures conformed to the 1964 Declaration of Helsinki and its subsequent amendments. Written informed consent in Arabic was obtained from every participant before enrolment. Data were de-identified and stored on access-restricted institutional servers.

### 2.3. Participants and Sampling

Eligible participants were adults aged 18 years or older with a clinician-documented diagnosis of cardiac arrhythmia (atrial fibrillation, atrial flutter, paroxysmal supraventricular tachycardia, or symptomatic ventricular ectopy) attending routine outpatient follow-up during the recruitment window. Each arrhythmia diagnosis was established by the treating cardiologist on the basis of 12-lead electrocardiography and, where clinically indicated, ambulatory rhythm monitoring, and was recorded in the electronic medical record. Exclusion criteria were acute clinical instability requiring immediate intervention, severe cognitive impairment or communication difficulty that precluded informed consent, and inability to complete the PSS-10 with standardised assistance. Consecutive sampling was applied, with every eligible patient on each clinic day approached by trained research assistants until the prespecified sample size was reached.

### 2.4. Sample Size

Sample size was determined by the requirements of the planned psychometric analysis. For factor analysis of a 10-item scale, a participant-to-item ratio of at least 10:1 is widely recommended, with ratios of 20:1 or higher preferred for stable solutions. A target of 282 participants provided a 28:1 ratio, generous for both exploratory and confirmatory factor analysis on split halves of approximately 141 participants each. For the multivariable logistic regression, simulation studies suggest that a minimum of 10 outcome events per candidate covariate yields approximately unbiased estimates; the achieved 69 high-stress events accommodated up to six covariates under this rule. Precision for reported effect sizes was quantified using 95% CIs rather than by an a priori power calculation, in keeping with current guidance for descriptive epidemiological studies. The study was designed to characterise correlates of perceived stress within this clinical population rather than to generate nationally representative prevalence estimates; sample size was therefore not derived from a population-extrapolation framework, and the precision of the estimated high-stress prevalence is reported as a 95% CI (Section 3.2).

### 2.5. Instrument

The questionnaire comprised three sections. Section A captured sociodemographic characteristics, namely age, sex, marital status, educational attainment, monthly household income, and current smoking status. Section B captured clinical characteristics covering arrhythmia subtype, time since diagnosis, self-reported information received about the condition, and the presence of T2DM, hypertension, hyperlipidaemia, and any prior cardiac surgical or ablation procedure. T2DM status was confirmed through review of the patient’s electronic medical record (documented diagnosis or current antidiabetic prescription); hypertension and hyperlipidaemia were verified against the medical record where possible, with self-report used only when documentation was incomplete. Section C contained the Arabic PSS-10, used with permission [28]. A previously validated Arabic version of the PSS-10 was adopted rather than undertaking a new translation; the cross-cultural adaptation and psychometric validation of this version are reported in the source publication [28]. Each item is scored from 0 (never) to 4 (very often), with items 4, 5, 7, and 8 reverse-scored, yielding a total score from 0 to 40 in which higher scores indicate greater perceived stress. The conventional cut-point of 20 or higher was used to define high perceived stress, consistent with prior cardiovascular literature [12,25].

Income insufficiency was operationalised as a self-reported monthly household income below 2400 Saudi Riyals (SAR), the sufficiency line published by the King Khaled Foundation [29]; because this threshold predates the study by several years, a sensitivity analysis was conducted using an inflation-adjusted threshold of 2750 SAR (Section 2.7).

### 2.6. Pilot Testing and Data Collection Procedure

The questionnaire was pilot-tested with 30 patients who met the eligibility criteria but were not included in the analytic sample. Comprehension, completion time (median 11 min), and item clarity were assessed. Minor wording adjustments to the sociodemographic items were endorsed by two bilingual cardiology pharmacists and a clinical psychologist familiar with PSS-10 use in Arabic. The pilot Cronbach’s α for the PSS-10 was 0.89.

For the main study, eligible patients were approached in a private clinic area after their scheduled consultation. Following written informed consent, participants completed the questionnaire either independently or with neutral, standardised assistance from research staff for participants with literacy limitations. Completed questionnaires were checked for completeness on the spot and entered into a password-protected REDCap database with double data entry for 10% of records. The English version of the full questionnaire is provided in Supplementary File S2.

### 2.7. Statistical Analysis

Analyses were performed in IBM SPSS Statistics version 27.0 (IBM Corp., Armonk, NY, USA) and AMOS version 27 for confirmatory factor analysis. Two-sided *p* values below 0.05 were considered statistically significant.

Continuous variables were summarised as mean (SD), and categorical variables as count and percentage. The PSS-10 distribution was inspected for normality using the Kolmogorov–Smirnov test together with skewness and kurtosis statistics; the score was approximately normally distributed (skewness 0.31, kurtosis −0.42). Group comparisons of mean PSS-10 scores were therefore conducted using independent-samples *t*-tests, with Cohen’s *d* as the effect-size measure. Associations between categorical variables and high perceived stress were tested with Pearson’s χ^2^, with Cramer’s *V* as the effect-size measure.

The primary multivariable analysis was a theoretically specified logistic regression with high perceived stress as the dependent variable. Covariates were selected a priori on the basis of stress-process theory and existing evidence rather than by univariate *p* value screening, comprising age (continuous), sex, marital status, education, income insufficiency, smoking status, time since diagnosis, T2DM, hypertension, and hyperlipidaemia. Multicollinearity was assessed using variance inflation factors (VIFs), with all values below 2.0. Because preliminary modelling produced unstable estimates for certain covariates with sparse cell counts, Firth’s penalised maximum likelihood was used as the estimation method, which produces approximately unbiased estimates and finite confidence intervals in the presence of separation or quasi-separation [30]. Linearity of the logit for age was confirmed by the Box-Tidwell test. Model performance was assessed with the Hosmer–Lemeshow goodness-of-fit test and the area under the receiver-operating-characteristic curve (AUROC).

Three sensitivity analyses were prespecified. The first re-fitted the model after excluding participants with missing covariate data; the second used the inflation-adjusted income threshold of 2750 SAR; the third used the continuous PSS-10 score as the outcome in an ordinary least-squares regression with the same covariates, in order to address the information loss associated with dichotomisation.

For the psychometric analysis, the sample was randomly split into two equal halves stratified by sex and T2DM status. Exploratory factor analysis (EFA) was conducted on the first half (*n* = 141) using maximum likelihood extraction with oblique (promax) rotation, given the theoretical expectation that the latent factors are correlated. Sampling adequacy was assessed using the Kaiser-Meyer-Olkin (KMO) measure and Bartlett’s test of sphericity. The number of factors was determined by parallel analysis, scree plot inspection, theoretical interpretability, and a minimum 10% incremental variance criterion, rather than by the Kaiser eigenvalue-greater-than-one rule alone. Confirmatory factor analysis (CFA) was performed on the second half (*n* = 141) in AMOS, with model fit evaluated against prespecified thresholds (RMSEA at or below 0.08; CFI at or above 0.95 with 0.90 acceptable; TLI at or above 0.95 with 0.90 acceptable; SRMR at or below 0.08) [31]. Internal consistency was quantified by Cronbach’s α for the full scale and for each factor.

## 3. Results

### 3.1. Participant Characteristics

Of 312 eligible patients approached, 282 (90.4%) consented and completed the questionnaire, with all 282 included in the analytic sample. Reasons for non-participation were declined consent (*n* = 18), incomplete PSS-10 (*n* = 8), and acute clinical event during the visit (*n* = 4). The sample was 59.6% male, with a mean age of 62.4 (SD 11.2) years. AF was the most frequent arrhythmia subtype (61.0%), followed by atrial flutter (16.7%), paroxysmal supraventricular tachycardia (13.5%), and symptomatic ventricular ectopy (8.9%). T2DM was present in 52.8% of participants, hyperlipidaemia in 53.9%, and hypertension in 40.4%. Income insufficiency, defined as monthly household income below 2400 SAR, was reported by 30.5% of participants. Participant characteristics are detailed in Table 1. Mean PSS-10 scores were similar across arrhythmia subtypes (atrial fibrillation 15.78, atrial flutter 15.22, paroxysmal supraventricular tachycardia 14.84, and ventricular ectopy 15.04; Table 1), indicating that perceived stress did not concentrate within any single arrhythmia category.

### 3.2. Distribution of Perceived Stress

The mean PSS-10 score was 15.5 (SD 9.1; range 0 to 40). High perceived stress (score of 20 or higher) was reported by 69 participants (24.5%; 95% CI, 19.7–29.9). The score distribution was approximately normal (skewness 0.31, kurtosis −0.42). Internal consistency of the Arabic PSS-10 was excellent (Cronbach’s α = 0.92; item-total correlations ranging from 0.58 to 0.76).

### 3.3. Bivariate Associations Between Participant Characteristics and Perceived Stress

Univariate comparisons of mean PSS-10 by sociodemographic and clinical strata are summarised in Table 1. Three associations reached statistical significance. Participants reporting income insufficiency had higher mean PSS-10 scores than those reporting sufficient income (18.20 (SD 10.04) versus 14.30 (SD 8.34); *t* = 3.41, *df* = 280, *p* = 0.001; *d* = 0.43). Participants with T2DM scored higher than those without (16.86 (SD 9.48) versus 13.91 (SD 8.30); *t* = 2.67, *df* = 280, *p* = 0.008; *d* = 0.33). Participants older than 60 years scored higher than those aged 60 years or younger (16.18 (SD 9.47) versus 14.37 (SD 8.26); *t* = 2.05, *df* = 280, *p* = 0.042; *d* = 0.20). The remaining covariates did not reach statistical significance, although the male versus female and hyperlipidaemia comparisons approached the conventional threshold (*p* = 0.068 and *p* = 0.102, respectively).

The pattern of bivariate associations with the binary high-stress outcome was consistent with the continuous-score analyses (Table 2). Income insufficiency (χ^2^ = 9.71, *df* = 1, *p* = 0.002; *V* = 0.19) and T2DM (χ^2^ = 11.43, *df* = 1, *p* < 0.001; *V* = 0.20) showed the strongest associations. Among participants reporting insufficient income, 34.9% (30 of 86) met the high-stress threshold, compared with 19.9% (39 of 196) among those reporting sufficient income. Among participants with T2DM, 32.2% (48 of 149) met the high-stress threshold, compared with 15.8% (21 of 133) among those without T2DM.

### 3.4. Multivariable Logistic Regression

Table 3 presents the theoretically specified multivariable logistic regression with Firth penalisation. After adjustment for age, sex, marital status, education, smoking status, time since diagnosis, hypertension, and hyperlipidaemia, two covariates remained independently associated with high perceived stress. Income insufficiency was associated with approximately twofold higher odds (aOR 2.09; 95% CI, 1.16–3.78; *p* = 0.014); T2DM with approximately 2.5-fold higher odds (aOR 2.51; 95% CI, 1.39–4.55; *p* = 0.002). Age and sex were not independently associated with high perceived stress in the adjusted model. VIFs for all covariates were below 1.4, indicating no problematic multicollinearity. The Hosmer–Lemeshow test indicated adequate model fit (χ^2^ = 6.84, *df* = 8, *p* = 0.554), and the model discriminated moderately between high-stress and low-stress participants (AUROC 0.71; 95% CI, 0.64–0.78).

The three prespecified sensitivity analyses produced consistent results. Restricting to complete cases (*n* = 274) yielded aORs of 2.06 (95% CI, 1.13–3.75) for income insufficiency and 2.49 (95% CI, 1.36–4.55) for T2DM. Using the inflation-adjusted income threshold of 2750 SAR yielded an aOR of 1.94 (95% CI, 1.10–3.42). Treating PSS-10 as a continuous outcome in linear regression produced coefficients of +3.62 points (95% CI, +1.31 to +5.93; *p* = 0.002) for income insufficiency and +2.78 points (95% CI, +0.62 to +4.94; *p* = 0.012) for T2DM.

### 3.5. Factor Structure of the Arabic PSS-10

**Figure 1:**
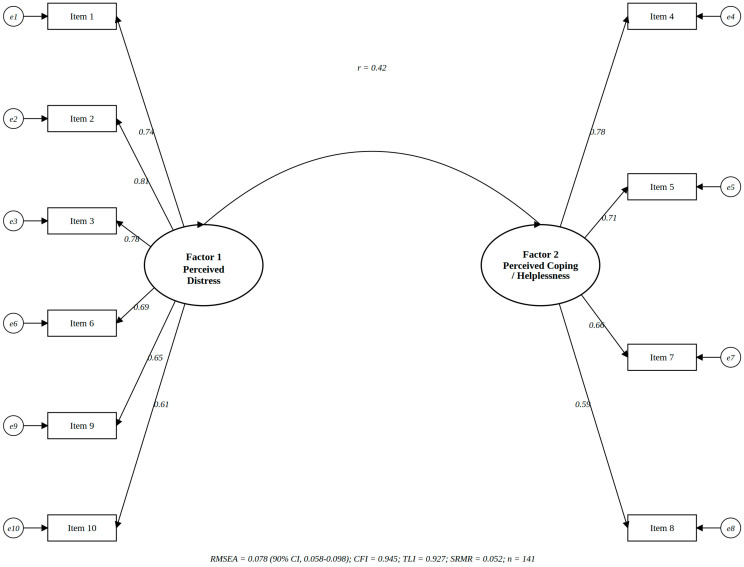
Two-factor confirmatory factor analysis (CFA) model of the Arabic Perceived Stress Scale-10 (PSS-10) on the holdout sample (n = 141). Standardised factor loadings are shown for all 10 items. Factor 1 (perceived distress) comprises the six negatively worded items (items 1, 2, 3, 6, 9, 10), with loadings ranging from 0.61 to 0.81. Factor 2 (perceived coping/helplessness) comprises the four reverse-scored items (items 4, 5, 7, 8), with loadings ranging from 0.59 to 0.78. The double-headed curved arrow between factors represents the inter-factor correlation (r = 0.42), which justified the use of oblique (promax) rotation in the exploratory factor analysis. Cross-loadings were modest (all < 0.30) and are omitted for clarity. Model fit indices on the holdout sample: RMSEA = 0.078 (90% CI, 0.058–0.098), CFI = 0.945, TLI = 0.927, SRMR = 0.052; the two-factor model fitted significantly better than a single-factor alternative (Δχ^2^ = 38.4, Δ*df* = 1, *p* < 0.001). Internal consistency: Cronbach’s α = 0.90 for Factor 1, α = 0.78 for Factor 2, α = 0.92 for the full scale. Abbreviations: CFA, confirmatory factor analysis; CFI, comparative fit index; PSS-10, 10-item Perceived Stress Scale; RMSEA, root mean square error of approximation; SRMR, standardised root mean square residual; TLI, Tucker–Lewis index.

The randomly split EFA sample (*n* = 141) showed excellent sampling adequacy (KMO = 0.91; Bartlett’s test of sphericity χ^2^ = 845.3, *df* = 45, *p* < 0.001). Parallel analysis supported retention of two factors, consistent with the theoretical structure of the PSS-10. Maximum likelihood extraction with promax rotation yielded a coherent two-factor solution accounting for 62.8% of common variance. Factor 1 (perceived distress) comprised the six negatively worded items, with primary loadings between 0.61 and 0.81. Factor 2 (perceived coping/helplessness) comprised the four reverse-scored items (items 4, 5, 7, and 8), with primary loadings between 0.59 and 0.78. Cross-loadings were modest (all below 0.30), and the factor correlation was 0.42, supporting the use of oblique rotation (Figure 1).

CFA on the holdout sample (*n* = 141) showed acceptable fit to the two-factor model: RMSEA = 0.078 (90% CI, 0.058–0.098), CFI = 0.945, TLI = 0.927, and SRMR = 0.052. The two-factor model fitted significantly better than a single-factor alternative (delta χ^2^ = 38.4, Δ*df* = 1, *p* < 0.001). Cronbach’s α was 0.90 for the perceived-distress subscale and 0.78 for the perceived-coping subscale.

## 4. Discussion

### 4.1. Principal Findings

Two correlates emerged as independently associated with high perceived stress after multivariable adjustment: income insufficiency and T2DM. The pattern was consistent. Roughly one in four of the 282 Saudi outpatients with cardiac arrhythmia met the high-stress threshold (Table 1), with each correlate conferring between two- and two-and-a-half-fold higher adjusted odds. Age and sex, modestly associated in unadjusted comparisons, did not retain independent significance. The Arabic PSS-10 showed a coherent two-factor structure with acceptable fit and high internal consistency, indicating that the total score is structurally interpretable in this population. Because the study was cross-sectional, these associations should be interpreted as concurrent correlations rather than as evidence that income insufficiency or T2DM temporally precedes, or causes, high perceived stress. To our knowledge, this is the first study to combine an evaluation of the latent structure of the Arabic PSS-10 with an analysis of the sociodemographic and clinical correlates of perceived stress in an Arab arrhythmia population; the mean PSS-10 score observed here is broadly consistent with the elevated stress previously reported in treated AF cohorts [12], while extending that work by identifying the subgroups in which the burden concentrates.

### 4.2. Income Insufficiency and Perceived Stress

The income-stress association was the most consistent finding across analyses, persisting in the binary outcome model (Table 3), in the inflation-adjusted sensitivity analysis, in the continuous-score regression, and in the complete-case analysis. A patient who must choose between a refilled antiarrhythmic prescription and a household necessity is not merely &quot;stressed&quot; in some generic sense, the predictability and controllability of daily life are themselves under threat. The pattern aligns with reviews documenting socioeconomic gradients in perceived stress and downstream health outcomes [17] and with Saudi epidemiological work showing income-related inequalities across cardiovascular and broader non-communicable disease prevalence [6,19]. Reverse causation, in which high perceived stress erodes work capacity and earning potential, remains plausible.

### 4.3. Type 2 Diabetes and Perceived Stress

Diabetes may shape generalised stress more broadly than its disease-specific emotional load alone would suggest. Roughly one-third of adults with T2DM experience clinically significant emotional distress related to self-management [32]. The adjusted association we observed (Table 3) is consistent with that literature, since self-monitoring, treatment adjustment, dietary management, and complication vigilance, taken together, constitute a continuous decision-making demand that may erode perceived control, with compounding effects when arrhythmia regimens add intricacy and recurrence uncertainty to the picture. Intervention work targeting diabetes-related distress has translated into improved glycaemic and self-management outcomes [33]; whether equivalent gains accrue from generalised stress reduction in arrhythmia-T2DM comorbidity remains an open question. Beyond this emotional load, T2DM adds a considerable cardiometabolic and therapeutic burden in the arrhythmia patient: overlapping regimens for glycaemic control, anticoagulation, rate or rhythm control, and lipid management compound treatment complexity and widen the scope for drug interactions, on top of the day-to-day self-monitoring these regimens demand. This accumulation of demands offers a plausible route by which diabetes amplifies perceived overload in this population.

### 4.4. Demographic Correlates

That age and sex showed modest unadjusted but no adjusted associations is itself informative (Table 3). Two interpretations merit consideration. The first is that age and sex may operate as proxies for differential exposure to economic strain and chronic disease burden, with their crude associations attenuated once those underlying determinants are introduced. The second is that the multivariable model has reduced precision relative to the bivariate analyses, particularly for variables with smaller cell counts. Still, either reading supports the same practical implication. Demographic stratification alone is unlikely to identify the patients most burdened by perceived stress in arrhythmia care, whereas income status and T2DM status did so more effectively in this sample.

### 4.5. Variables Without Significant Association

Marital status, education, smoking status, time since diagnosis, hypertension, and self-reported information about the condition did not show significant associations with high perceived stress, in either the bivariate analyses (Table 2) or the multivariable model (Table 3). Two readings are possible. The substantive interpretation is that these factors carry less stress weight than economic strain and diabetes self-management; the methodological interpretation is that single-item or coarsely categorised exposures may compress meaningful variability. Marital status as a binary, for instance, conflates supportive marital contexts with unsupportive ones, and &quot;information about the condition&quot; as a single item does not capture health literacy, the quality of counselling received, or the patient’s capacity to translate information into self-management. The hyperlipidaemia association approached but did not reach significance and may reflect cumulative cardiometabolic burden, although confirmation requires a larger sample.

### 4.6. Psychometric Findings

The split-sample EFA-CFA design supports the structural adequacy of the Arabic PSS-10 total score in a cardiac population, with two latent factors (perceived distress and perceived coping/helplessness) and acceptable fit on the holdout sample (Figure 1). The psychometric evaluation was undertaken to support the primary clinical analysis rather than as a standalone validation of the instrument, and the two components are reported together for that reason. Convergent and discriminant validity were not examined, since no second measure of stress or emotional distress was administered (Section 4.8). The factor correlation of 0.42 indicates that the dimensions are related but not redundant. One possibility raised by this analysis is that different determinants map differentially onto the dimensions: income insufficiency may primarily increase the distress dimension, while T2DM may raise both. Item-level mediation analyses in larger samples could test this hypothesis directly. The borderline RMSEA of 0.078 sits within conventional thresholds [31] but warrants independent replication in other Arab cardiac populations before subscale scores are used in clinical decision-making.

### 4.7. Clinical Implementation in Cardiology Practice

The findings suggest a candidate operational pathway for the arrhythmia clinic that would require prospective evaluation before it could be recommended for routine adoption. First, the PSS-10 is short enough to fit visit flow without disrupting it. Median completion time was 11 min in pilot testing, and the high-stress proportion of roughly one in four justifies systematic rather than selective administration. Second, two routinely collected variables, namely self-reported income sufficiency and documented T2DM status, identify the highest-risk subgroup at the level of the registration desk before the cardiology consultation begins, without requiring additional assessment instruments. Third, a positive screen calls for a referral pathway already available in most tertiary settings, in which the cardiology team initiates onward connection to financial-counselling services, structured diabetes-distress support, social-work consultation, and where appropriate cardiac rehabilitation linkage. Fourth, the screening and onward referral fits naturally with the clinical pharmacist or arrhythmia clinic nurse rather than the cardiologist. The pathway should not be confused with an evidence-based intervention; trial evaluation of integrated screen-and-refer packages is a precondition for guideline endorsement [34].

### 4.8. Strengths and Limitations

The principal strengths are theory-driven covariate selection, a priori multivariable model specification, the use of Firth penalisation to address sparse-data instability, the prespecified sensitivity analyses, and the split-sample psychometric design. Examination of the latent structure and internal consistency of the Arabic PSS-10 in a cardiac population, in which these properties had not previously been reported, adds methodological value beyond the substantive findings.

Several limitations should be considered. The cross-sectional design precludes causal inference and temporal sequence; whether income insufficiency and T2DM precede high perceived stress, follow it, or are reciprocally reinforcing cannot be determined. Recruitment from a single tertiary outpatient cardiology service in Alkharj limits generalisability; the findings may not transfer cleanly to inpatient cardiology units, to primary care, or to other Saudi regions, and the high consent rate of 90.4% reduces but does not eliminate selection bias. Hypertension and hyperlipidaemia status relied partly on self-report, which may have introduced misclassification, although T2DM was confirmed by medical record review. Several constructs were not measured here, including depressive symptoms, anxiety, perceived social support, and arrhythmia symptom severity. The sociodemographic and clinical items were study-developed and not independently validated as a standalone instrument. Because the questionnaire carried no second measure of stress or emotional distress, the convergent and discriminant validity of the Arabic PSS-10 could not be examined here. The psychometric analysis is accordingly confined to internal structure and reliability, and it does not constitute a full validation of the instrument in cardiac populations, which remains a task for future work. Dichotomising perceived stress at the conventional threshold improves clinical interpretability but reduces statistical power; the continuous-score sensitivity analysis was reassuring on this point but does not eliminate the concern. Finally, the income threshold derives from a 2017 reference document, and although the inflation-adjusted sensitivity analysis showed a consistent association, contemporary household-budget data would strengthen the operationalisation in future work.

### 4.9. Implications for Future Research

Four directions follow directly from these results. Longitudinal designs are needed to test whether changes in income status or diabetes management predict subsequent changes in perceived stress, and whether perceived stress, in turn, predicts arrhythmia-related clinical outcomes; candidate endpoints include symptom burden, hospitalisation, medication adherence, and post-ablation recurrence. Finer-grained socioeconomic measures (income-to-needs ratio, debt, cost-related medication non-adherence, employment instability) would identify which economic mechanisms carry the strongest stress weight. Expanded psychosocial covariates would permit formal mediation and moderation analyses consistent with stress-process theory [16]. Intervention trials should test integrated pathways combining PSS-10 screening with financial-navigation and T2DM-focused coping support, evaluating both psychological endpoints and clinical endpoints (adherence, symptom control, healthcare utilisation).

## 5. Conclusions

Two correlates were independently associated with high perceived stress in this Saudi outpatient arrhythmia cohort: income insufficiency and T2DM. The association was robust. It retained its association after adjustment for demographic and clinical covariates, while age and sex did not, and it was reproduced across all three prespecified sensitivity analyses without material attenuation in the effect estimates. The Arabic PSS-10 showed a coherent two-factor structure and high internal consistency, and its brief format makes it a practical candidate for stress screening in cardiology, pending confirmation of its measurement properties in independent samples. Brief stress screening, integrated diabetes-distress support, financial-navigation referral, and structured social-work consultation should be evaluated prospectively as components of routine arrhythmia care.

## Figures and Tables

**Table 1:** Sociodemographic and clinical characteristics of the study sample and bivariate associations with mean Perceived Stress Scale-10 (PSS-10) score (N = 282).

Characteristic	*n* (%)	Mean PSS-10 (SD)	*t* (*df*)	*p* Value	Cohen’s *d*
Sociodemographic					
Age > 60 years	162 (57.4)	16.18 (9.47)	2.05 (280)	*p* = 0.042	0.20
Age ≤ 60 years	120 (42.6)	14.37 (8.26)	Ref.	Ref.	Ref.
Male sex	168 (59.6)	16.10 (9.20)	1.83 (280)	*p* = 0.068	0.16
Married	236 (83.7)	15.42 (9.05)	0.62 (280)	*p* = 0.537	0.07
Higher education	142 (50.4)	14.98 (8.71)	1.27 (280)	*p* = 0.206	0.11
Income insufficiency	86 (30.5)	18.20 (10.04)	3.41 (280)	*p* = 0.001	0.43
Current smoker	64 (22.7)	16.05 (9.42)	0.62 (280)	*p* = 0.538	0.08
Clinical					
Atrial fibrillation	172 (61.0)	15.78 (9.18)	0.96 (280)	*p* = 0.337	0.11
Atrial flutter	47 (16.7)	15.22 (9.41)			
PSVT	38 (13.5)	14.84 (8.65)			
Ventricular ectopy	25 (8.9)	15.04 (9.12)			Ref.
Time since diagnosis ≥ 5 years	128 (45.4)	15.74 (9.18)	0.41 (280)	*p* = 0.682	0.05
Type 2 diabetes mellitus	149 (52.8)	16.86 (9.48)	2.67 (280)	*p* = 0.008	0.33
Hypertension	114 (40.4)	16.12 (9.41)	0.96 (280)	*p* = 0.335	0.10
Hyperlipidaemia	152 (53.9)	16.45 (9.30)	1.64 (280)	*p* = 0.102	0.20

Data are presented as n (%) for the participant column and mean (SD) for the PSS-10 column. Group comparisons of mean PSS-10 use independent-samples *t*-tests. The reference category for each binary covariate is the complementary group (e.g., age > 60 years vs. ≤60 years), shown as ‘Ref.’ in the test-statistic columns. Empty cells in the test-statistic columns indicate that no group comparison applies for that row (e.g., non-AF subtypes within the multi-category arrhythmia variable, where the test compares AF vs. non-AF combined). Bold typeface denotes statistically significant associations at *p* < 0.05. Cohen’s *d* effect-size benchmarks: small 0.20, medium 0.50, large 0.80. Abbreviations: PSS-10, 10-item Perceived Stress Scale; PSVT, paroxysmal supraventricular tachycardia; SD, standard deviation; df, degrees of freedom.

**Table 2:** Bivariate associations between sociodemographic and clinical characteristics and high perceived stress (PSS-10 score ≥ 20) (N = 282).

Characteristic	High Stress, *n*/*N* (%)	χ^2^ (*df*)	*p* Value	Cramer’s *V*
Age > 60 years	44/162 (27.2)	2.18 (1)	*p* = 0.140	0.09
Male sex	43/168 (25.6)	0.34 (1)	*p* = 0.561	0.04
Married	57/236 (24.2)	0.13 (1)	*p* = 0.722	0.02
Higher education	31/142 (21.8)	1.06 (1)	*p* = 0.304	0.06
Income insufficiency	30/86 (34.9)	9.71 (1)	*p* = 0.002	0.19
Current smoker	17/64 (26.6)	0.18 (1)	*p* = 0.671	0.03
Time since diagnosis ≥ 5 years	33/128 (25.8)	0.21 (1)	*p* = 0.649	0.03
Type 2 diabetes mellitus	48/149 (32.2)	11.43 (1)	*p* < 0.001	0.20
Hypertension	33/114 (28.9)	1.69 (1)	*p* = 0.194	0.08
Hyperlipidaemia	44/152 (28.9)	2.84 (1)	*p* = 0.092	0.10

High perceived stress is defined as a PSS-10 score ≥ 20, consistent with prior the cardiovascular literature. The reference category for each binary covariate is the complementary group. Pearson’s χ^2^ test (with continuity correction where appropriate) was used for all comparisons. Bold typeface denotes statistically significant associations at *p* < 0.05. Cramer’s *V* effect-size benchmarks: small 0.10, medium 0.30, large 0.50. Abbreviations: PSS-10, 10-item Perceived Stress Scale; df, degrees of freedom.

**Table 3:** Theoretically specified multivariable Firth-penalised logistic regression for high perceived stress (PSS-10 score ≥ 20) and prespecified sensitivity analyses (N = 282).

Covariate	aOR	95% CI	*p* Value
Primary multivariable model			
Age (per year)	1.02	0.99–1.04	*p* = 0.124
Male sex	1.18	0.66–2.10	*p* = 0.582
Married	0.92	0.45–1.86	*p* = 0.814
Higher education	0.74	0.42–1.31	*p* = 0.305
Income insufficiency	2.09	1.16–3.78	*p* = 0.014
Current smoker	1.29	0.66–2.51	*p* = 0.462
Time since diagnosis ≥ 5 years	1.07	0.61–1.88	*p* = 0.810
Type 2 diabetes mellitus	2.51	1.39–4.55	*p* = 0.002
Hypertension	1.43	0.79–2.59	*p* = 0.240
Hyperlipidaemia	1.62	0.92–2.86	*p* = 0.094
Sensitivity analyses (key exposures only)			
Complete-case (*n* = 274), income insufficiency	2.06	1.13–3.75	*p* = 0.018
Complete-case (*n* = 274), T2DM	2.49	1.36–4.55	*p* = 0.003
Inflation-adjusted threshold (2750 SAR), income	1.94	1.10–3.42	*p* = 0.022
Continuous PSS-10 (β, points), income	+3.62	+1.31 to +5.93	*p* = 0.002
Continuous PSS-10 (β, points), T2DM	+2.78	+0.62 to +4.94	*p* = 0.012

The primary multivariable model is a Firth-penalised logistic regression with high perceived stress (PSS-10 score ≥ 20) as the dependent variable. Covariates were specified a priori on the basis of stress-process theory and existing evidence. The reference category for each binary covariate is the complementary group. Variance inflation factors for all covariates were below 1.4. Model performance: Hosmer–Lemeshow χ^2^ = 6.84, *df* = 8, *p* = 0.554; AUROC 0.71 (95% CI, 0.64–0.78). Sensitivity analyses retained the full covariate set; only point estimates for the two key exposures are shown for compactness. The continuous-outcome rows report linear-regression β coefficients (in PSS-10 score points) rather than odds ratios. Bold typeface denotes statistically significant associations at *p* < 0.05. Abbreviations: aOR, adjusted odds ratio; AUROC, area under the receiver-operating-characteristic curve; CI, confidence interval; PSS-10, 10-item Perceived Stress Scale; SAR, Saudi Riyals; T2DM, type 2 diabetes mellitus; df, degrees of freedom.

## Data Availability

The de-identified participant data and the data dictionary supporting the reported results are available from the corresponding author upon reasonable request. Data are not publicly archived because the dataset includes information that could permit re-identification of participants in this single-centre cohort. Data sharing is subject to approval by the institutional ethics committee and to completion of a data-use agreement.

## Supplementary Materials

The following supporting information can be downloaded at: https://www.mdpi.com/article/10.3390/healthcare14142197/s1, File S1: STROBE Statement, checklist of items that should be included in reports of cross-sectional studies; File S2: English version of the full study questionnaire (sociodemographic, clinical, and PSS-10 items). Refs. [24,25,27,28] are cited in the Supplementary Material file.

## Abbreviations

The following abbreviations are used in this manuscript:

AF	Atrial fibrillation
aOR	Adjusted odds ratio
AUROC	Area under the receiver-operating-characteristic curve
CFA	Confirmatory factor analysis
CFI	Comparative fit index
CI	Confidence interval
*df*	Degrees of freedom
EFA	Exploratory factor analysis
KMO	Kaiser-Meyer-Olkin
PSS-10	10-item Perceived Stress Scale
RMSEA	Root mean square error of approximation
SAR	Saudi Riyals
SD	Standard deviation
SRMR	Standardised root mean square residual
STROBE	Strengthening the Reporting of Observational Studies in Epidemiology
T2DM	Type 2 diabetes mellitus
TLI	Tucker–Lewis index
VIF	Variance inflation factor
