# Supplementary material for: Income Insufficiency, Type 2 Diabetes, and Perceived Stress in Cardiac Arrhythmia Outpatients: A Cross-Sectional Study
Source: Healthcare (Basel). 2026 Jul 20;14(14):2197. doi: 10.3390/healthcare14142197 (PMC13409788; doi:10.3390/healthcare14142197)
Supplement: Supplementary file 1 [file healthcare-14-02197-s001.zip › 03_Supplementary_File_S1_STROBE_Checklist.pdf]

Supplementary File S1.

STROBE Statement — checklist of items that should be included in reports of cross-sectional studies

Reference: von Elm E, Altman DG, Egger M, Pocock SJ, Gotzsche PC, Vandenbroucke JP. The Strengthening the Reporting of Observational Studies in Epidemiology (STROBE) statement: guidelines for reporting observational studies. *Ann Intern Med*. 2007;147(8):573-77.

Section	Item No.	Recommendation	Reported on Page / Section
**Title and Abstract**	1	(a) Indicate the study's design with a commonly used term in the title or the abstract	Title page; Abstract
		(b) Provide in the abstract an informative and balanced summary of what was done and what was found	Abstract (Background, Methods, Results, Conclusions)
**Introduction**			
Background/rationale	2	Explain the scientific background and rationale for the investigation being reported	Section 1 (Introduction), paragraphs 1-6
Objectives	3	State specific objectives, including any prespecified hypotheses	Section 1 (Introduction), paragraph 7
**Methods**			
Study design	4	Present key elements of study design early in the paper	Section 2.1 (Study design and setting)
Setting	5	Describe the setting, locations, and relevant dates, including periods of recruitment, exposure, follow-up, and data collection	Section 2.1 (Study design and setting); Section 2.6 (Pilot testing and data collection procedure)
Participants	6	(a) Give the eligibility criteria, and the sources and methods of selection of participants	Section 2.3 (Participants and sampling)
Variables	7	Clearly define all outcomes, exposures, predictors, potential confounders, and effect modifiers. Give diagnostic criteria, if applicable	Section 2.5 (Instrument); Section 2.7 (Statistical analysis)
Data sources / measurement	8	For each variable of interest, give sources of data and details of methods of assessment (measurement). Describe comparability of assessment methods if there is more than one group	Section 2.5 (Instrument); Section 2.6 (Pilot testing)
Bias	9	Describe any efforts to address potential sources of bias	Section 2.6 (Pilot testing); Section 4.8 (Strengths and limitations)
Study size	10	Explain how the study size was arrived at	Section 2.4 (Sample size)
Quantitative variables	11	Explain how quantitative variables were handled in the analyses. If applicable, describe which groupings were chosen and why	Section 2.7 (Statistical analysis)
Statistical methods	12	(a) Describe all statistical methods, including those used to control for confounding	Section 2.7 (Statistical analysis)

		(b) Describe any methods used to examine subgroups and interactions	Section 2.7 (Statistical analysis), sensitivity analyses
		(c) Explain how missing data were addressed	Section 2.7 (Statistical analysis), complete-case sensitivity analysis
		(d) If applicable, describe analytical methods taking account of sampling strategy	Not applicable (consecutive sampling)
		(e) Describe any sensitivity analyses	Section 2.7 (Statistical analysis), three prespecified sensitivity analyses
**Results**			
Participants	13	(a) Report numbers of individuals at each stage of study (e.g., numbers potentially eligible, examined for eligibility, confirmed eligible, included in the study, completing follow-up, and analysed)	Section 3.1 (Participant characteristics)
		(b) Give reasons for non-participation at each stage	Section 3.1 (Participant characteristics)
		(c) Consider use of a flow diagram	Reasons for non-participation reported in prose (consort-style flow diagram available on request)
Descriptive data	14	(a) Give characteristics of study participants and information on exposures and potential confounders	Section 3.1; Table 1
		(b) Indicate number of participants with missing data for each variable of interest	Section 3.1 (8 incomplete PSS-10, 4 acute event excluded)
Outcome data	15	Report numbers of outcome events or summary measures	Section 3.2 (Distribution of perceived stress); Table 1
Main results	16	(a) Give unadjusted estimates and, if applicable, confounder-adjusted estimates and their precision (e.g., 95% confidence interval). Make clear which confounders were adjusted for and why they were included	Section 3.3 (Bivariate); Section 3.4 (Multivariable); Tables 1-3
		(b) Report category boundaries when continuous variables were categorized	Section 2.5 (PSS-10 cut-point); Section 2.7 (income threshold)
		(c) If relevant, consider translating estimates of relative risk into absolute risk for a meaningful time period	Section 3.4 (proportions in high-stress group reported)
Other analyses	17	Report other analyses done — e.g., analyses of subgroups and interactions, and sensitivity analyses	Section 3.4 (three sensitivity analyses); Table 3
**Discussion**			
Key results	18	Summarise key results with reference to study objectives	Section 4.1 (Principal findings)
Limitations	19	Discuss limitations of the study, taking into account sources of potential bias or imprecision. Discuss both direction and magnitude of any potential bias	Section 4.8 (Strengths and limitations)
Interpretation	20	Give a cautious overall interpretation of results considering objectives, limitations, multiplicity of analyses, results from similar studies, and other relevant evidence	Sections 4.2-4.7 (subsection-by-subsection interpretation)

Generalisability	21	Discuss the generalisability (external validity) of the study results	Section 4.8 (Strengths and limitations)
**Other Information**			
Funding	22	Give the source of funding and the role of the funders for the present study and, if applicable, for the original study on which the present article is based	Declarations (Funding)

Note: An Explanation and Elaboration article discusses each checklist item and gives methodological background and published examples of transparent reporting. The STROBE checklist is best used in conjunction with this article (freely available on the Web sites of PLoS Medicine at *http://www.plosmedicine.org/*, Annals of Internal Medicine at *http://www.annals.org/*, and Epidemiology at *http://www.epidem.com/*). Information on the STROBE Initiative is available at *http://www.strobe-statement.org*.
