# Supplementary material for: Income Insufficiency, Type 2 Diabetes, and Perceived Stress in Cardiac Arrhythmia Outpatients: A Cross-Sectional Study
Source: Healthcare (Basel). 2026 Jul 20;14(14):2197. doi: 10.3390/healthcare14142197 (PMC13409788; doi:10.3390/healthcare14142197)
Supplement: Supplementary file 1 [file healthcare-14-02197-s001.zip › 06_Supplementary_File_S2_Questionnaire.pdf]

Supplementary File S2.

English-language version of the study questionnaire (sociodemographic, clinical, and Perceived Stress Scale-10 items)

Manuscript: Perceived stress and its sociodemographic and clinical correlates in adults with cardiac arrhythmia attending a tertiary outpatient clinic in Saudi Arabia: a cross-sectional study

Description. This file presents the English version of the structured questionnaire administered to study participants. The original instrument was administered in Arabic. Section A captured sociodemographic characteristics, Section B captured clinical characteristics, and Section C contained the 10-item Perceived Stress Scale (PSS-10). The PSS-10 was administered using the previously validated Arabic translation by Chaaya and colleagues (BMC Psychiatry 2010; 10:111), used with permission. The English wording of the PSS-10 items shown here corresponds to the original instrument by Cohen, Kamarck, and Mermelstein (J Health Soc Behav 1983; 24:385-96). The questionnaire was pilot-tested with 30 patients (median completion time 11 minutes) before main-study administration.

Administration notes. Participants completed the questionnaire either independently or with neutral, standardised assistance from research staff for participants with literacy limitations. Section B clinical items were verified against the participant's electronic medical record where possible (T2DM status was confirmed for all participants by record review; hypertension and hyperlipidaemia status used self-report only when documentation was incomplete). Completed questionnaires were checked for completeness on the spot and entered into a password-protected REDCap database with double data entry for 10% of records.

Section A. Sociodemographic Characteristics

Please answer the following questions about yourself. Your responses will be kept strictly confidential and used only for research purposes.

A1. Age (in completed years): ____________ years

A2. Sex (please mark one):

☐ Male

☐ Female

A3. Marital status (please mark one):

☐ Single (never married)

☐ Married

☐ Divorced

☐ Widowed

☐ Separated

A4. Highest level of education completed (please mark one):

☐ No formal education

☐ Primary school (grades 1-6)

☐ Intermediate school (grades 7-9)

☐ Secondary school (grades 10-12)

☐ Diploma or technical certificate

☐ Bachelor's degree

☐ Postgraduate degree (Master's or Doctorate)

A5. Approximate monthly household income (Saudi Riyals, SAR; please mark one):

☐ Less than 2,400 SAR

☐ 2,400 to 4,999 SAR

☐ 5,000 to 9,999 SAR

☐ 10,000 to 19,999 SAR

☐ 20,000 SAR or more

☐ Prefer not to answer

A5a. Considering your typical monthly expenses, would you say your household income is sufficient to cover your basic needs (housing, food, healthcare, utilities)?

☐ Yes, sufficient

☐ No, insufficient

A6. Current smoking status (please mark one):

☐ Never smoker

☐ Former smoker (quit ≥ 12 months ago)

☐ Current smoker (any tobacco product, including cigarettes, shisha, or e-cigarettes, used in the past 30 days)

Section B. Clinical Characteristics

This section asks about your heart rhythm condition and other medical conditions. Items will be cross-checked with your medical record where possible.

B1. Type of cardiac arrhythmia diagnosed by your cardiologist (please mark one; to be confirmed against medical record):

- ☐ Atrial fibrillation (AF)
- ☐ Atrial flutter
- ☐ Paroxysmal supraventricular tachycardia (PSVT)
- ☐ Symptomatic ventricular ectopy / premature ventricular contractions
- ☐ Other (please specify): ____________________

B2. Approximately how long ago were you first diagnosed with your heart rhythm condition?

- ☐ Less than 1 year ago
- ☐ 1 to 2 years ago
- ☐ More than 2 to 5 years ago
- ☐ More than 5 to 10 years ago
- ☐ More than 10 years ago

B3. How would you rate the amount of information you have received about your heart rhythm condition from your healthcare team?

- ☐ I received a great deal of information and feel well informed
- ☐ I received some information and feel reasonably informed
- ☐ I received only basic information and feel under-informed
- ☐ I received very little information and feel uninformed

B4. Have you been diagnosed with any of the following conditions? (please mark all that apply; will be verified against medical record where possible):

- ☐ Type 2 diabetes mellitus (T2DM)
- ☐ Hypertension (high blood pressure)
- ☐ Hyperlipidaemia (high cholesterol)
- ☐ Coronary artery disease
- ☐ Heart failure
- ☐ Previous stroke or transient ischaemic attack
- ☐ Chronic kidney disease
- ☐ None of the above

B5. Have you ever had any of the following cardiac procedures? (please mark all that apply):

☐ Catheter ablation for arrhythmia

☐ Pacemaker implantation

☐ Implantable cardioverter-defibrillator (ICD) implantation

☐ Cardioversion (electrical or chemical)

☐ Coronary artery bypass grafting (CABG)

☐ Percutaneous coronary intervention (PCI / stenting)

☐ Heart valve surgery

☐ None of the above

B6. Please list the cardiac medications you are currently taking (or check 'See medical record' if you prefer the research team to extract this from your file):

☐ See medical record

Or list here: ____

____

Section C. Perceived Stress Scale-10 (PSS-10)

Source. The 10-item Perceived Stress Scale was developed by Cohen, Kamarck, and Mermelstein (1983). The Arabic version administered to participants in this study was the validated translation by Chaaya, Osman, Naassan, and Mahfoud (BMC Psychiatry 2010; 10:111), used with permission. The English item wording shown below is the original Cohen et al. (1983) instrument and is reproduced here for the reader's reference.

Instructions. The questions in this scale ask you about your feelings and thoughts during the last month. In each case, you will be asked to indicate by circling how often you felt or thought a certain way. Although some of the questions are similar, there are differences between them and you should treat each one as a separate question. The best approach is to answer each question fairly quickly. That is, do not try to count up the number of times you felt a particular way; rather indicate the alternative that seems like a reasonable estimate.

Response options for each item:

Score	Response
0	Never
1	Almost never
2	Sometimes
3	Fairly often
4	Very often

PSS-10 Items

Item	Statement	0	1	2	3	4
1	In the last month, how often have you been upset because of something that happened unexpectedly?	☐	☐	☐	☐	☐
2	In the last month, how often have you felt that you were unable to control the important things in your life?	☐	☐	☐	☐	☐
3	In the last month, how often have you felt nervous and stressed?	☐	☐	☐	☐	☐
4 *	In the last month, how often have you felt confident about your ability to handle your personal problems?	☐	☐	☐	☐	☐
5 *	In the last month, how often have you felt that things were going your way?	☐	☐	☐	☐	☐
6	In the last month, how often have you found that you could not cope with all the things that you had to do?	☐	☐	☐	☐	☐
7 *	In the last month, how often have you been able to control irritations in your life?	☐	☐	☐	☐	☐
8 *	In the last month, how often have you felt that you were on top of things?	☐	☐	☐	☐	☐

9	In the last month, how often have you been angered because of things that happened that were outside of your control?	☐	☐	☐	☐	☐
10	In the last month, how often have you felt difficulties were piling up so high that you could not overcome them?	☐	☐	☐	☐	☐

* Reverse-scored items (items 4, 5, 7, and 8). For these four items, raw responses are reverse-coded before summation: 0 → 4, 1 → 3, 2 → 2, 3 → 1, 4 → 0.

Scoring of the PSS-10

PSS-10 scores are obtained by reversing the four positively worded items (items 4, 5, 7, and 8) and then summing all 10 items. The total score ranges from 0 to 40, with higher scores indicating greater perceived stress. In the present study, high perceived stress was operationally defined as a total score of 20 or higher, consistent with prior cardiovascular literature.

Subscale composition (as identified in the present study)

Factor 1, Perceived Distress: Items 1, 2, 3, 6, 9, and 10 (the six negatively worded items). Standardised loadings in the present study ranged from 0.61 to 0.81. Cronbach's α = 0.90.

Factor 2, Perceived Coping/Helplessness: Items 4, 5, 7, and 8 (the four reverse-scored items). Standardised loadings in the present study ranged from 0.59 to 0.78. Cronbach's α = 0.78.

Internal consistency in the present study: The full 10-item Arabic PSS-10 demonstrated excellent internal consistency in the present sample (Cronbach's α = 0.92; item-total correlations 0.58 to 0.76).

References for the Instrument

1. Cohen S, Kamarck T, Mermelstein R. A global measure of perceived stress. J Health Soc Behav. 1983;24(4):385-96. doi:10.2307/2136404.
2. Chaaya M, Osman H, Naassan G, Mahfoud Z. Validation of the Arabic version of the Cohen Perceived Stress Scale (PSS-10) among pregnant and postpartum women. BMC Psychiatry. 2010;10:111. doi:10.1186/1471-244X-10-111.
3. Roberti JW, Harrington LN, Storch EA. Further psychometric support for the 10-item version of the Perceived Stress Scale. J Coll Couns. 2006;9(2):135-47. doi:10.1002/j.2161-1882.2006.tb00100.x.

Permissions and acknowledgements

The PSS-10 is freely available for non-commercial research use, with attribution to Cohen, Kamarck, and Mermelstein (1983). The Arabic translation by Chaaya and colleagues (2010) was used with the authors' permission. The questionnaire reproduced in this supplementary file is provided solely for transparent reporting of the study instrument and is not intended for redistribution outside the context of this manuscript.
